# Vitamin D Deficiency in Pregnant Women and Their Neonates

**DOI:** 10.5539/gjhs.v8n9p83

**Published:** 2015-10-29

**Authors:** Maryam Abbasian, Reza Chaman, Mohammad Amiri, Mohammad Esmaeil Ajami, Tohid Jafari-Koshki, Hossein Rohani, Seyed Mahmood Taghavi-Shahri, Erfan Sadeghi, Mehdi Raei

**Affiliations:** 1 School of Medicine, Shahroud University of Medical Sciences, Shahroud, Iran; 2 Department of Community Medicine, School of Medicine, Yasuj University of Medical Sciences, Yasuj, Iran; 3 Department of Public Health, School of Health, Shahroud University of Medical Sciences, Shahroud, Iran; 4 School of Nursing and Midwifery, Shahroud University of Medical Sciences, Shahroud, Iran; 5 Department of Biostatistics, School of Health, Sabzevar University of Medical Sciences, Sabzevar, Iran; 6 Department of Statistics and Epidemiology, School of Health, Tabriz University of Medical Sciences, Tabriz, Iran; 7 Department of Health Education and Promotion, School of Health, Isfahan University of Medical Sciences, Isfahan, Iran; 8 Department of Biostatistics, School of Health, Kermanshah University of Medical Sciences, Kermanshah, Iran; 9 Department of Biostatistics, Faculty of Medicine, Shiraz University of Medical Sciences, Shiraz, Iran; 10 Clinical Research Development Center, Qom University of Medical Sciences, Qom, Iran; 11 Department of Biostatistics, School of Health, Isfahan University of Medical Sciences, Isfahan, Iran

**Keywords:** vitamin D deficiency, calcium deficiency, pregnant women, neonate

## Abstract

**Background and Objective:**

Vitamin D deficiency during pregnancy is a worldwide problem. Studies have reported prevalence ranged 18-84% in pregnant women. Receiving adequate calcium and vitamin D during pregnancy period is necessary for calcium homeostasis, fetal growth and bone mineralization. This study was aimed to determine the prevalence of vitamin D deficiency in pregnant women and their neonates in Shahroud city in the northeast Iran.

**Methods:**

In this cross-sectional study, 284 pregnant women and their neonates referred to Fatemiyeh Hospital of Shahroud were included. Blood samples of mothers and umbilical cords were collected during the delivery and were sent to laboratory in order to measure calcium and 25-hydroxy vitamin D.

**Findings:**

Amounts of Vitamin D insufficiency (20-30 ng/mL) and deficiency (<20 ng/mL) in (mothers, neonates) were found to be (60.2%, 48.9%) and (1.1%, 2.5%) respectively. Calcium deficiency (<8.5 mg/dL) was present in 33.5% of mothers and 25% of neonates. There was a weak correlation between maternal serum and cord blood 25-hydroxy vitamin D (r=0.12, p=0.053).

**Conclusion:**

More than half of the mothers and their neonates had some degrees of vitamin D deficiency. It is recommended to evaluate the nutritional status of vitamin D in pregnant women along with public health interventions to be carried out.

## 1. Introduction

Vitamin D is a steroidal prohormone, the active type which plays a significant role in absorption of calcium and phosphate. It reaches the body through skin synthesis via ultraviolet rays when the sun exposure is adequate and can be ingested through food. Through influencing the absorption of calcium in the intestinal tissues and other effects on bone and other tissues of the body, the metabolites of this vitamin have key roles in regulating the metabolism of minerals (Mulligan et al., 2010). During pregnancy, fundamental changes occur in calcium and vitamin D metabolism (Kovacs, 2001). Requirement for calcium increases in the third trimester of pregnancy so that in the final stages of pregnancy, about 30g of the calcium for the fetal skeleton is created from the maternal skeleton and through hormonal intervention (Sabour et al., 2006). So, it is vital to receive adequate vitamin D and calcium during the pregnancy for the fetal homeostasis, bone growth and mineral development (Cavalier et al., 2008).

The optimal vitamin D concentration in pregnancy and newborns is still disputed and unknown (Wagner, & Greer 2008; Holick et al., 2011; Marshall et al., 2013). It is generally accepted that a level of 11 ng/mL in newborns is sufficient to prevent rickets and a serum level of >30 ng/mL is necessary to control secondary hyperparathyroidism as well as increase calcium absorption. Levels of >30 ng/mL are accepted as ideal, and levels <20 ng/mL are considered deficient (Holick et al., 2011).

While research on the implications of vitamin D deficiency in the immediate perinatal period is ongoing, it would be sensible to institute interventions that could narrow the insufficiency period. The best way of achieving this would be to supply adequate vitamin D to mothers during pregnancy and the lactation period (Shakiba et al., 2014).

Vitamin D deficiency brings about obvious damages to the growth and development of fetal bones (Cavalier et al., 2008). It can lead to osteomalacia in pregnancy, skeletal abnormalities and fetal mineral bone acquisition in childhood, and influences the neonatal skeletal development, bone size and impaired bone mass accrual (Viljakainen et al., 2010; Dawodu & Akinbi 2013). Extraskeletal consequences of vitamin D deficiency in pregnancy include potentially increased risks of gestational diabetes mellitus, preeclampsia and maternal bacterial vaginosis but not delivery by caesarean section. It increases the risk of intrauterine growth retardation, low birth weight and early-onset sepsis in infants. In addition, sufficient vitamin D concentration in pregnancy is supposed to have a positive influence on mother’s and child’s immune system (McCarthy et al., 2009; Soheilykhah et al., 2010; Aghajafari et al., 2013, Dawodu & Akinbi, 2013; Pooraziz, 2015).

Adequate vitamin D supplementation during pregnancy may be helpful to prevent early-onset sepsis in term neonates (Cetinkaya et al., 2014). Risk factors of vitamin D deficiency include receiving insufficient vitamins, low intake of fortified food, life style, seasonal changes, skin color, low compliance of supplementation, inadequate sun exposure, premature birth, body covering, overweight and living at high latitude (Hagenau et al., 2009; De Ronne & De Schepper, 2013).

High prevalence of vitamin D deficiency has been reported among pregnant women and neonates in different countries. Extensive research indicates that the prevalence of vitamin D deficiency varies from 18-84% depending on factors such as ethnicity, region, culture and customs in different countries (Kovacs, 2001). In the north of Canada, 46% of healthy mothers and 36% of their babies, in England 18% of pregnant women and 70% of their babies, in Northern India 84%, and in New Zealand 61% of pregnant women, in the Netherlands, 60-84% of pregnant women had vitamin D deficiency (Pawley, & Bishop, 2004; Judkins & Eagleton, 2005; Sachan et al., 2005; Ward, 2005; van der Meer et al., 2006). A number of studies have been conducted on estimating vitamin D deficiency in Iran and various estimates have been reported for pregnant women and neonates (Bassir et al., 2001; Hashemipour et al., 2004; Maghboli et al., 2006; Maghbooli et al., 2007). These indicate that vitamin D deficiency is frequent among Iranian pregnant women and it can be inferred that majority of pregnant women suffer from, at least, from vitamin D insufficiency. Note that these estimates are based on different categorization of vitamin D levels as the deficiency in each study refers to various ranges such as < 20, <25, and <35 ng/mL.

These studies have used different cut off points for assessing vitamin D deficiency. Considering the different opinions, a scale has been suggested dividing vitamin D concentrations into deficiency (<50 nmol/L), insufficiency (50–80 nmol/L) and optimal values (>80 nmol/L) (Hollis et al., 2011). One review summarized evidence from studies that evaluated cut off point for serum 25(OH)D concentrations in relation to bone mineral density and other consequences of vitamin D deficiency and showed that the most advantageous serum concentrations of 25(OH)D begin at 75 nmol/L (30 ng/mL) (Bischoff-Ferrari et al., 2006).

With regard to the fact that very few studies have investigated the vitamin D deficiency among pregnant women and their neonates in Iran, this study aimed to investigate the status of this vitamin and its serum level among pregnant women and babies in Shahroud, Northeast of Iran.

## 2. Methods

This cross-sectional study was carried out on 284 pregnant women referred to Fatemiyeh Hospital of Shahroud from winter 2012 to spring 2013.

The sample size was determined to be 280 subjects in the basis of other studies in which the prevalence of vitamin D deficiency was estimated to be about 58% in mothers and 46% in neonates.

The sampling procedure continued until the required number of participants consented to take part in the study. Inclusion criteria consisted of all new admissions to antepartum, emergency, labor and delivery wards of the Fatemiyeh hospital. Exclusion criteria were taking drugs, which could influence vitamin D and calcium metabolism and renal, hepatic, endocrine and metabolic bone diseases. The participants were interviewed and the researchers completed the checklist forms, which included items on demographic data, pregnancy status, history of osteoporosis and bone fracture and history of drug use during pregnancy. The heights and weights of the participants were also measured and recorded.

In the delivery room, maternal and cord blood samples were taken from mothers and the neonates, the serum of which was immediately isolated in the hospital laboratory and sent to the central laboratory for further tests. The samples were kept at the temperature of -70°C until they were tested. Then, the serum was sent to laboratory for measuring 25-hydroxy vitamin D. ELISA method and the kits produced by IDS Company in England were used to measure 25-hydroxy vitamin D and calorimetry and concave therapeutic kits were used to measure Ca. Deficiency criteria of 25-hydroxy vitamin D and Ca were considered the same in both mothers and neonates. The calcium serum ranging 8.5-11 mg/dL was regarded as normal and <8.5 mg/dL as deficient. Likewise for vitamin D, ranges <20 ng/mL were regarded as deficient, 20-30 ng/mL as insufficient and >30 ng/mL as sufficient (Bischoff-Ferrari et al., 2006).

A written informed consent was obtained from all participants and they were informed that participation was anonymous and voluntary. They were also ensured that the results would be confidential and advantageous to them.

The collected data was statistically analyzed by SPSS version 18.0 for Windows (SPSS Inc, Chicago, Illinois, USA). Descriptive statistical techniques were used to determine the prevalence and the means. Pearson correlation test was used to examine the correlation between the variables. p-values less than 0.05 was considered to be statistically significant.

## 3. Results

A total of 284 pregnant women were enrolled in which, 57.7% of them lived in cities and 3.9% were illiterate. The mean age of participants was 26.62±5.32 years and 1.8% of them had delivery before 37 weeks of gestation (Table 1).

**Table 1:** Socio-demographic and reproductive characteristics of participants

Variables	Amount
Maternal age (year)	26.62±5.32
Maternal education (illiterate)	11(3.9%)
Maternal job (employed)	15(5.3%)
Location (city)	164(57.7%)
Infant’s gender (boy)	140(49.3%)
Infant’s birth weight (grams)	3184.79±496.07
Infant’s gestational age at birth (<37w)	5(1.8%)
Delivery type (Cesarean)	161(56.7%)
Maternal BMI (kg/m^2^)^a^	37.09±144.19

The mean level of maternal and cord Ca were respectively 9.06±1.22 and 9.4±1.2 mg/dL. Normal level of Ca was found in 61.6% of mothers and 63.4% of neonates (Table 2) and which were statistically associated (r=0.25, p<0.001).

**Table 2:** The status of Ca and vitamin D in pregnant women and infants

	Frequency	Percent	95% CI
**Maternal Ca**			
Normal	175	61.6	55.9-67.28
Low	95	33.5	27.9-38.9
High	14	4.9	2.4-7.4
Total	284	100	-
**Infant’s Ca**			
Normal	180	63.4	57.8-68.9
Low	71	25	20.0-30.0
High	33	11.6	7.9-15.4
Total	284	100	-
**Maternal Vitamin D**			
Deficient	3	1.1	0.27-2.8
Insufficient	171	60.2	54.4-65.8
Sufficient	110	38.7	33.2-44.5
Total	284	100	-
**Infant’s Vitamin D**			
Deficient	7	2.5	1.1-4.8
Insufficient	139	48.9	43.2-57.5
Sufficient	138	48.6	42.8-54.4
Total	284	100	-

The mean level of vitamin D in mothers and neonates were respectively 28.5±11.75 and 29.69±11.94 ng/mL. Vitamin D was sufficient in 38.7% of mothers while 60.2% of them had vitamin D insufficiency (<30 ng/mL) and only 48.6% of the neonates had sufficient levels of vitamin D (Table 2). Cord blood vitamin D revealed to have a weak significant relationship with maternal serum level of vitamin D (r=0.115, p=0.053).

No association was found between neither age and vitamin D status (r=0.0008, p=0.8), nor age and serum Ca level (r=–0.023, p=0.69) of mothers. There was also no significant relationship between maternal Ca and serum level of vitamin D (r=0.07, p=0.23). Moreover, serum vitamin D level of pregnant women was not associated to neonatal birth weight (r=0.018, p=0.76). In pregnant women, Ca and vitamin D level had no significant relationship with their gravidity number (r=-0.024, p=0.69; r=0.055, p=0.35).

## 4. Discussion

The findings of this study indicate that vitamion D insufficiency is present in more than half of the mothers and neonates, (60.2% of mothers and 48.9% of neonates). Various researches show that the prevalence of vitamin D deficiency is ranging 5-50% in mothers and 10-56% in neonates (Kovacs, 2001; Vandevijvere et al., 2012; Choi, Kim et al., 2015; Soltirovska Salamon et al., 2015; Vinkhuyzen et al., 2015). In a study on 50 mothers and neonates, Weiler et al. found that more than half of the mothers and more than a third of neonates had low vitamin D levels and therefore are suffering from vitamin D deficiency (Weiler et al., 2005).

Bondar and colleagues in a study on 400 pregnant women and their neonates (200 blacks and 200 whites) reported some degrees of vitamin D deficiency. They reported insufficient vitamin D in 54.1% of black and 42.1% of white women. Moreover, 46.8% of black mothers’ neonates and 56.4% of white mothers’ neonates were vitamin D insufficient (Bodnar et al., 2007). This is consistent with the findings of the present study. A study by Maghbooli and colleagues also confirms the deficiency of vitamin D in 66.8% of the mothers (Maghbooli et al., 2007). In a study on 88 pregnant women in Isfahan, Salek et al. also reported a 26.1% of vitamin D deficiency among mothers and 53.4% among neonates (Salek et al., 2008). Moreover, a similar study in Tehran reported the prevalence of vitamin D deficiency to be from 9.5% to 57.6% (Ainy, Ghazi et al., 2006). A study in Zanjan also indicated that the prevalence of vitamin D deficiency exists in 86% of the mothers and 75% of their neonates in winter, while in summer these proportions falls to 46% and 35%, respectively (Kazemi, Sharifi et al., 2009). High prevalence of vitamin D deficiency has been reported in women in different countries which is in line with the findings of our study. In Northern India, New Zealand and the Netherlands, respectively, 42%, 61%, and 60-84% of pregnant women and in north Canada, 46% of mothers and 36% of their babies, are vitamin D deficient (Judkins, & Eagleton, 2005; Sachan et al., 2005; Ward, 2005; van der Meer et al., 2006).

Our findings indicated that the mean level of vitamin D in mothers and neonates were 28.5±11.75 and 29.69±11.94 ng/mL, respectively. In Viljakainen et al.’s study, the mean level of vitamin D level was 41.0±13.6 and 50.7±14.9 nmol/L during the first trimester and in the umbilical cord, respectively (Viljakainen et al., 2010). In Dovnik et al.’s study, the average vitamin D concentration in the September group neonates was 54.3±25.2 nmol/L, and in the December group neonates was 33.3±18.6 nmol/L. Also, the average vitamin D concentration in women who took vitamin-D-containing nutritional supplements during pregnancy was 68.9±27.0 nmol/L in September, and for those who did not take these supplements it was 46.5±20.3 nmol/L (Dovnik, Mujezinović et al., 2014). In Bassir et al.’s study the mean cord vitamin D concentration was 4.94±9.4 nmol/L while for infants of mother with hypovitaminosis D, it was almost undetectable (1.2±1.2 nmol/L) (Bassir et al., 2001).

In our study, no significant relationship was found between maternal serum level of vitamin D and of Ca similar to the study of Maghboli et al, in which there was a significant correlation between maternal and cord blood vitamin D (r=0.7, p<0.001) but a weak non-significant relationship between maternal serum and cord blood vitamin D. (r= 0.115, p =0.53) (Maghboli et al., 2006).

In a study performed by Nicolaidou et al. in Greece, 19.5% of mothers and 8.1% of neonates had vitamin D <10 ng/mL. They also found a strong relationship between maternal and cord blood vitamin D (r=0.62, p<0.001) (Hollis et al., 2011). The present study also showed a low level in 1.1% of mothers and 2.5% of neonates.

In our study, cord blood vitamin D showed a weak significant association with maternal serum level of vitamin D. Similar to us, in Maghbooli et al.’s study, a significant correlation was found between maternal and cord blood serum concentrations of vitamin D (Maghbooli et al., 2007). In Dovnik et al.’s study maternal and cord blood vitamin D values correlated strongly and neonatal vitamin D values were approximately 20 nmol/L higher than maternal values in both groups (Dovnik et al., 2014).

Aly et al. and Sachan et al. reported a similar strong correlation between maternal and fetal vitamin D values (Sachan et al., 2005; Aly et al., 2013).

In our study serum vitamin D level of pregnant women had no significant association with neonatal birth weight. Similar to us, in Maghbooli et al.’s study, there was no significant correlation between maternal vitamin D deficiency with the newborn’s weight, height, or head circumference (Maghbooli et al., 2007).

In follow-up assessments by Shefras et al., a significant association between vitamin D received and head circumference was found (Shefras, & Farquharson, 1996). Also, Marya et al. reported that infants of mothers who received vitamin D had a greater head circumference compared with infants whose mothers did not receive vitamin D (Marya et al., 1988). The cut-off point for vitamin D deficiency may have an influence on detectable changes in pregnancy outcomes.

Vitamin D deficiency occurs rather commonly among healthy pregnant women and newborns. Since it plays a role in calcium and phosphor metabolism essential for bone health and in the physiopathology of some autoimmune diseases, it seems important to come up with solutions to prevent vitamin D deficiency (De Ronne & De Schepper, 2013). To provide adequate levels of vitamin D to newborns, the American Academy of Pediatrics recommended universal oral vitamin D supplementation at 400 IU/day for all breastfeeding infants born to vitamin D deficient mothers and this should be continued till the age of 6 years. In cases of dark skin the dose should be 600 IU/day (Wagner, & Greer, 2008). A healthy life style with outdoor activities and associated sun exposure along with intake of fortified nutrition should be advised (Dovnik et al., 2014). Shakiba et al. reported clinical implications of four-month lag for attaining vitamin D sufficiency in 90% of infants (Shakiba et al., 2014).

## 5. Conclusion

According to our findings, more than half of the mothers and their neonates had some degrees of vitamin D deficiency and with regard to the important effects of this vitamin D deficiency on the health of both pregnant women and their neonates, we suggest the investigation of vitamin D nutritional status in pregnant women as well as public health interventions.

A major limitation to our study is that we were unable to monitor sun exposure in our infants and mothers. As it is customary in Iran to prevent the exposure of infants to the sun in the early months of life, we assumed that sun exposure had limited effects. Therefore, mothers included in our study have probably had prior limited sun exposure and were all vitamin D deficient, and it is unlikely that they would have changed their lifestyles during the study period.
